# What regulatory agencies like HEC, PM&DC can do to help improve quality and standard of Pakistani Biomedical Journals

**DOI:** 10.12669/pjms.332.12857

**Published:** 2017

**Authors:** Shaukat Ali Jawaid, Masood Jawaid

**Affiliations:** 1 Shaukat Ali Jawaid Chief Editor, Pakistan Journal of Medical Sciences, Karachi - Pakistan; 2 Masood Jawaid Associate Editor Pakistan Journal of Medical Sciences, Karachi - Pakistan

Journalology has now emerged as an important discipline in itself with numerous sub-specialties like Peer Review, Scientific Misconduct and Publication Ethics. There have been numerous developments in the information technology as well which while on one hand has made the job of Editors easy but it has also brought with it too many problems under which the editors have to work.^1^ The world of scientific publishing and Medical Writing is also witnessing lot of developments with numerous innovations and it is difficult even for a full time editor to keep pace with these developments, what to talk of physicians who are functioning as Editors as a part time hobby. It took a long time for the medical profession to realize that one may be a good Physician or good Surgeon but he or she cannot be a good Teacher. Hence, now many medical institutions have started Certificate Courses in Teaching while some have also initiated Master’s Course in Health Professionals Education and these have also become mandatory in some countries. Likewise, it may take another ten to fifteen years for the medical profession to realize that one can be a Good Physician and Good Surgeon but cannot be a Good Editor. Medical Journalism is a combination of art and science and one has to learn all this.

A vast majority of biomedical journals published by various medical institutions and medial universities in the developing world Pakistan being no exception, have head of these institutions as Chief Editor of these journals. Most of them having no practical working experience, can become Patrons but it will be in the interest of their institutions as well that they acquire the services of those healthcare professionals who are conversant with this art to assume the responsibilities of editing the journals. It is extremely important to have some minimum dedicated staff including an Editor. He or she can be a doctor, nurse, medical journalist and even other technical experts who can then coordinate with other members of the team to produce a quality journal. Currently a large number of biomedical journals are recognized by Pakistan Medical & Dental Council as well as Higher Education Commission, Government of Pakistan. However, many of them suffer from many deficiencies and shortcomings. While recognizing these journals, those responsible in these regulatory agencies, perhaps did not look at it carefully while evaluating these journals or they are themselves not aware of it. What is further surprising is the fact that they are not even prepared to seek help and assistance from the professional bodies like Pakistan Association of Medical Editors (PAME) which has offered its services time and again without any obligation. President of PAME was once notified as ex-officio member of the Journals Committee by the PM&DC but then no meeting has taken place since then.^2^

**Fig.1:**
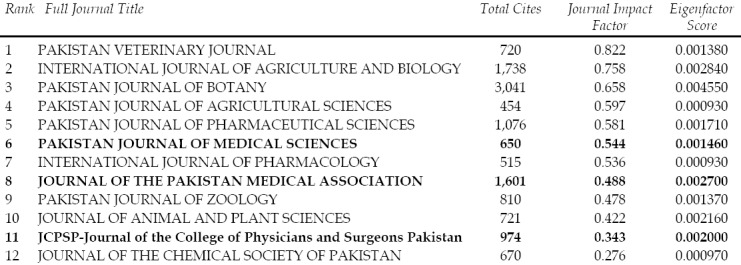
Pakistani Biomedical Journals covered by ISI Thomson Reuters: WoS”

Higher Education Commission does organize meetings from time to time asking the journals it has recognized to make presentation about their performance and plans for future development. It also offers financial grant to the journals depending on their category but this is not going to help improve standard of Pakistani biomedical journals. We have time and again suggested that instead of financial grant, let the HEC provide some services which the journals cannot afford themselves.^3^ For example, if it can acquire software for making XML files required by PubMed Central, a number of Pakistani biomedical journals will be able to move to PubMed Central thus improving their visibility to the international readers. Thus these journals will also automatically become visible on PubMed/Medline as well. HEC as well as PM&DC can also organize training courses for Editors, Hands on workshops not only for Editors but authors and reviewers as well, something PAME has been doing for the last many years with its own limited resources. HEC working in collaboration with PM&DC and PAME can also identify about a dozen good quality journals which has the potential but are not yet indexed in international databases like Medline and ISI Thompson Reuters, Science Citation Index Expanded (SCIE) known for its Impact Factor. Then a committee of expert editors can be formed to help, guide editors of these selected journals so that they can be covered by these international databases which will also increase the number of Pakistani biomedical journals having Impact Factor. These journals should be given a road map in writing how to proceed further. Their progress can then be monitored and evaluated after every six months. If we plan even today in 2017, the results will become visible after three to four years. Similarly one can go on adding two to three journals to this list of selected journals every year. Those journals which are successful in getting included in these databases can be removed from this list and a few more journals with potential of making to these databases should be included and asked to move on the same path. At present twelve Pakistani journals which includes just three medical journals have Impact Factor.^4^ (Fig.1) Impact Factor is of course not the only but one of the criteria’s to judge the standard of a journal.^5^-^6^ While the HEC and medical universities insist for the authors to get their manuscripts published in Impact Factor journals, it has added to their worries and also created problems for the editors of Pakistani medical journals which enjoy Impact Factor. They are under too much pressure from the authors but these journals have their own financial as well as human resource limitations. Starting from formatting, initial internal review, screening for plagiarism and then external review takes lot of time and there is no short cut. Finding good quality reviewers is an uphill task. We in Pakistan Journal of Medial Sciences have provided fast track processing facility but it is available only to a selected few who have to appear in CPSP exam for Fellowship or they require it for their M. Phil or PhD Thesis, Dissertations. We do not encourage fast track processing unless it is extremely essential but then end up annoying a large number of authors who are keen to get their manuscripts processed on fast track. One hopes that the concerned authorities will take note of it and come up with some initiatives to help the authors as well as Editors. Let them learn from the Journals Commission formed by the Ministry of Health, Government of Iran.^7^ This commission has a number of professional editors as members and they help journals in designing websites, getting them indexed in international database, provide them facility of XML filing etc., and not financial grant something which our Higher Education Commission has been doing. Unless the HEC changes its policy, we cannot expect to have more journals earning Impact Factor or becoming visible on databases like Medline, SCIE, Web of Sciences, and ISI Thompson Reuter. We on our part feel happy of having helped quite a few other Editor Colleagues and medical journals in different ways. We have also instituted Dr. Maqbool H. Jafary Training Scholarship for training of junior editors in the memory of our Founder Chief Editor who played a commendable role in promoting the art of medical writing and science of scientific publishing in Pakistan. This week long intensive course also carries scholarship of Rs. 25,000/- The first candidate has already completed this course last year and we hope to train atleast one junior editor every year.

However, it is quite painful to report that the meeting organized by HEC at its regional Center at Karachi on April 7, 2017 was just an apology. It also reflected the lack of vision, foresight as well as proper sense of direction on the part of the organizers. The participants were not told about the agenda and they did not know what is going to be discussed till the last minute. A few professional medical editors among the participants who are affiliated with the journals with Impact Factor which are also covered by all the international databases did point out the viewpoint of Pakistan Association Medical Editors. (PAME). They were much better informed and knowledgeable about Journalology and Medical Journalism, problems and difficulties faced by Editors of peer reviewed journals as compared to the experts arranged by the HEC. Little do the organizers realize that such halfhearted measures and un-professional attitude brings bad name to the Higher Education Commission. It is high time that the higher administration of HEC which monitors universities and other institutions of higher education in Pakistan also starts looking inward and monitors the functioning and performance of its various divisions. What they have been doing for the last so many years, what has been their accomplishments so far if any, and what are their future plans? Instead of policing, let the Quality Assurance Division of HEC entrusted with the evaluation of biomedical journals do some soul searching and start acting as facilitators. Instead of wasting HEC funds on such meetings without any clear objectives, it needs to put in some efforts in the positive direction so that more and more Pakistani biomedical journals become visible on international databases like PubMed/Medline, PubMed Central and earn Impact Factor.

Some of the professional medical editors in the meeting did point out the short comings and also questioned the criteria of evaluation of the journals used by HEC which lacks transparency but the organizers failed to give any satisfactory answer with the result that there could not be any fruitful interactive discussion. Perhaps it has not yet dawned upon them that we are now living in an era of professionalism. When some of these professional editors were asked about the feedback about the meeting, they opined that “it was just waste of time”.
